# Impact of family history on oncological outcomes in primary therapy for localized prostate cancer patients: a systematic review and meta-analysis

**DOI:** 10.1038/s41391-021-00329-0

**Published:** 2021-02-15

**Authors:** Fumihiko Urabe, Shoji Kimura, Shutaro Yamamoto, Kojiro Tashiro, Takahiro Kimura, Shin Egawa

**Affiliations:** Department of Urology, The Jikei University School of Medicine, Tokyo, Japan

**Keywords:** Prostate cancer, Outcomes research

## Abstract

**Background:**

The influence of family history on oncological outcomes of prostate cancer remains controversial. We conducted a systematic literature review and meta-analysis to investigate the impact of family history of localized prostate cancer on oncological outcomes.

**Methods:**

On May 2020, we systematically searched MEDLINE, the Cochrane library, and Scopus for studies that compared patients who had localized prostate cancer with or without a positive family history of prostate cancer. Our aim was to evaluate the association of family history with biochemical recurrence-free survival, cancer-specific survival, and overall survival by means of a multivariate Cox regression analysis.

**Results:**

Eleven studies with 39,716 patients were included in the systematic review, and eight studies with 33,027 patients for the meta-analysis. A positive family history was not associated with worse biochemical recurrence-free survival (pooled HR: 0.96; 95% CI: 0.79–1.17) or cancer-specific survival (pooled HR: 1.1; 95% CI: 0.52–2.35). Subgroup analyses showed no association between positive family history and poor biochemical recurrence-free survival in prostate cancer patients treated with radical prostatectomy (pooled HR: 0.99; 95% CI: 0.76–1.31) or radiation therapy (pooled HR: 0.93; 95% CI: 0.67–1.30).

**Conclusions:**

This meta-analysis indicated that family history of prostate cancer does not increase the risk of biochemical recurrence or cancer-specific mortality in localized prostate cancer patients.

## Introduction

Prostate cancer (PCa) is the second most frequent male cancer in worldwide [1], and a family history of PCa is a well-known risk factor for the development of this disease. The individual relative risk of a PCa diagnosis is doubled in first-degree male relatives of PCa patients [2], and 10–20% of PCa patients are reported to have a positive family history among their first-degree relatives [3]. Although numerous studies have focused on the clinicopathological features associated with family history of PCa [4–6], clinical observation has shown no substantial differences between PCa patients with family history and those without, except that family history is associated with younger age at diagnosis [2]. Recent findings point to genetic factors that underlie familial aggregations of PCa; genetic differences between patients with and without family history of PCa can be based on single-nucleotide polymorphisms or germline mutations, including HOXB13, BRCA1, BRCA2, and MLH1, which are associated with an increased risk of familial PCa [7–11].

The influence of family history on oncological outcomes is less clear. For example, Kupelian et al. reported a negative association between family history and survival outcomes [12], while Westerman et al. stated that family history of PCa was significantly correlated with improved oncological outcomes [13]. Clearly, further work is needed to fully understand the prognostic value of family history in oncological outcomes for patients with localized PCa. Such an understanding could contribute to improved clinical practices and facilitate the process of shared decision-making with patients.

Therefore, in this study, we hypothesized that the family history would influence the oncological outcomes in localized PCa. To verify this hypothesis, we conducted a systematic review and meta-analysis of the literature to investigate and summarize the association between family history and oncological outcomes in localized PCa.

## Material and methods

### Literature search strategy

We performed this systematic review and meta-analysis according to the Preferred Reporting Items for Systematic Reviews and Meta-analysis statement [14]. The protocol was preregistered in the International Prospective Register of Systematic Reviews database (PROSPERO: CRD42020188782). We searched MEDLINE, Cochrane Library, and Scopus databases on May 11, 2020 for studies published through April 2020 investigating the impact of family history on oncologic outcomes in localized PCa patients. To expand the data search, we attempted to identify unpublished studies in ISRCTN registry ClinicalTrials.gov, World Health Organization International Clinical Trials Registry Platform, OpenGrey, and Database of Abstracts of Reviews of Effects on the same day. The first screening was based on study title and abstract, after which the full-text papers were assessed for eligibility. All extracted data were crosschecked to ensure their appropriateness based on full-text review. Two authors (FU and SK) independently carried out this process, and all discrepancies were generally resolved by consensus or by referring to the senior author (SE). The following string terms were used in this study: “prostate cancer” and “family history”, and “biochemical recurrence” OR “oncological outcome” OR “overall survival”. Biochemical recurrence-free survival (BCRFS) was our primary outcome of interest, and cancer-specific survival (CSS), and overall survival (OS) were secondary outcomes. EndNote X8 was used as a bibliography management software.

### Selection criteria

Studies were considered eligible if they compared localized PCa patients (patients) with a positive family history (exposure) to those without family history (comparison) to assess the effect of family history on BCRFS, CSS, and OS (outcomes), utilizing univariate and multivariable Cox regression analysis in cohort studies. Articles published in languages other than English, reviews, commentaries, case series were excluded. If there were multiple articles published by same group using similar cohorts, either the more recent or the higher quality publication was included.

### Data extraction

Two authors (FU and SK) independently extracted data from all the eligible studies. The following characteristics were contained: first author’s name, publication year, country in which patients were enrolled, recruitment period, number of patients, age, prostate-specific antigen (PSA), primary therapy, follow-up duration, and family history of PCa. Hazard ratios (HRs) and 95% confidence intervals were determined for family history of PCa associated with each of the oncological outcomes. All discrepancies regarding data extraction were generally resolved by consensus or by a meeting with the other authors (SY, KT, TK, and SE).

### Statistical analysis

A forest plot was used to assess HRs from the multivariable analyses of individual studies and obtained a summary HR for the effect of family history in localized PCa on cancer progression and mortality. Studies with general logistic regression, log-rank, Kaplan–Meier, univariable Cox proportional hazard regression analyses were not included in this meta-analysis. In cases where only the HR and *P* value were reported, we calculated the 95% CI [15, 16]. Statistical heterogeneity among the studies was assessed using the Cochrane Q test and I-square test. *P* value < 0.05 in the Cochrane Q test and a ratio >50% in the I-square test were defined as statistically significant. The summary HRs, and the 95% CI were calculated using random effects models. Fixed effect models were used to calculate pooled HRs for non-heterogeneous results [17–19]. Publication bias was determined using funnel plots. The level of statistical significance was set at *P* values < 0.05. Statistical analyses were all conducted using the Stata/MP, version 14.2 (Stata Corp., College Station, TX, USA)

### Quality assessment

We assessed the quality of included studies using The Newcastle-Ottawa Scale [20] based on the Cochrane Handbook for systematic reviews [21, 22]. The scale is allocated according to three domains: population selection (maximum of four stars), comparability of the group (maximum of two stars), and ascertainment of exposure (maximum of three stars). The total scale ranges from 0 to 9. The main confounders were identified as the important prognosis factors of BCRFS, CSS, and OS. The presence of confounders was determined by a review of reported data and consensus. We identified those studies with scores higher than 6 as “high-quality” choices.

### Risk of bias

We assessed the risk-of-bias of the included studies according to the Cochrane Handbook for Systematic Reviews of Interventions for including non-randomized studies [22]. The confounding factors were selected as the most common prognostic factors at the time of diagnosis. The articles were therefore reviewed based on the adjustment for the effect of age, PSA, tumor staging, and grading according to the investigated outcomes. The Risk Of Bias In Non-randomized Studies of Interventions tool was used for BCRFS and CSS analyzed [23]. The risk of bias of each study was assessed by two authors (FU and SK), independently. Discrepancies were resolved by consultation with the senior author (SE). The results of risk-of-bias assessment were summarized in Supplementary Tables (Tables S1, S2).

## Results

### Study selection

**Fig. 1:**
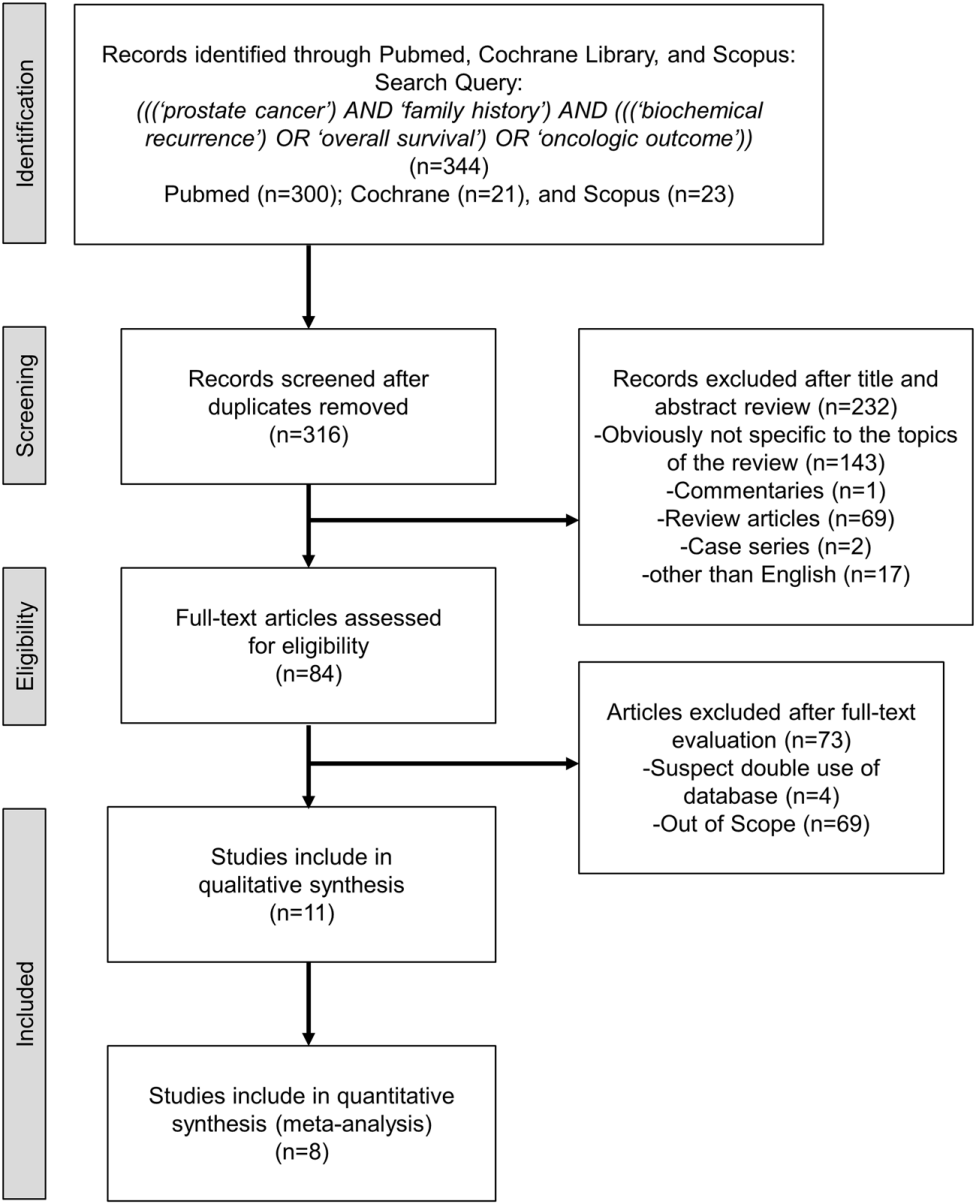
Study selection process. Flow chart for the process of article selection to analyze the impact of family history on oncological outcomes.

Overall, 316 articles were identified from the search query, of which 232 articles were removed after title and abstract assessment (Fig. 1). No unpublished study was identified by Gray Literature search. An additional 73 articles were excluded after full-text evaluation. Finally, the remaining 11 articles were included in the systematic review [6, 13, 24–32] and 8 articles in the qualitative meta-analysis [13, 25–28, 30–32].

### Study population

The studies’ characteristics and patients’ clinical data are summarized in Tables 1, 2, respectively. The 11 studies comprise 39,716 treatment-naive PCa patients. The examined populations were Northern American in nine studies, European in one, and Asian in one. All studies were published between 1999 and 2017. The definition of family history was the presence of one or more first-degree relatives in ten studies; the strict definition including hereditary PCa and sporadic PCa was used in the remaining study. The number of patients with family history was provided in all 11 studies and accounted for 10,471 of all 39,716 patients (26.4%). Median or mean follow-up ranged from 40 months to 9.9 years. In most studies, the median or mean age of patients with family history was younger than in those without. The value of median or mean PSA was similar between PCa patients with and without family history. Primary therapies were radical prostatectomy (RP) or radiation therapy (RT). Only a univariate Cox regression analysis was performed in three studies, which were excluded from meta-analysis. A multivariate Cox regression analysis was applied to the remaining eight studies. Meta-analyses were conducted for BCRFS and CSS. Only two studies evaluated the association of OS with family history: Westerman et al. reported the rate of 10-year OS was greater in patients with family history than in those without, but Bagshaw et al. found no significant difference in OS between patients with or without family history [13, 31].

**Table 1:** The studies’ characteristics of all included articles.

First author of study and [Ref.]	Country	Recruitment period	Study design	Level of evidence	Definition of FH	Total	FH		NOS
							Yes	No	
Hanus et al. [24]	US	1987–1996	Cohort, retrospective	2B	One or more first-degree relatives	1164	148	1016	6
Ray et al. [25]	US	1983–2001	Cohort, retrospective	2B	One or more first-degree relatives	536	97	439	7
Lee et al. [26]	US	1989–2000	Cohort, retrospective	2B	One or more first-degree relatives	556	103	453	7
Spangler et al. [27]	US	1995–2002	Cohort, retrospective	2B	One or more first-degree relatives	506	179	327	6
Roehl et al. [28]	US	1983–2003	Cohort, retrospective	2B	One or more first-degree relatives	1186	Sibiling pairs (*n* = 141) Hereditary (*n* = 114) High density family member (*n* = 37)	968	7
Kupelian et al. [29]	US	1986–2002	Cohort, retrospective	2B	One or more first-degree relatives	4112	659	3453	7
Peters et al. [30]	US	1992–2005	Cohort, retrospective	2B	One or more first-degree relatives	1737	187	1550	7
Bagshaw et al. [31]	US	1989–2007	Cohort, retrospective	2B	One or more first-degree relatives	1711	386	1325	7
Westerman et al. [13]	US	1987–2010	Cohort, retrospective	2B	One or more first-degree relatives	16,472	5323	11,149	7
Thalgott et al. [32]	Germany	1994–2010	Cohort, retrospective	2B	HPC: subgroup of familial PCa with a likelihood of genetic predisposioton SPC: two or more brothers aged at least 60 y and negative anamnesces for PCa	11,130 (FPC + SPC *n* = 2775)	HPC (*n* = 807) FPC (*n* = 2251)	Non-FPC (*n* = 8072) SPC (*n* = 524)	7
Lee et al. [6]	Korea	2006–2015	Cohort, retrospective	2B	One or more first-degree relatives	606	39	567	6

*FH* family history, *PCa* prostate cancer, *HPC* hereditary prostate cancer, *FPC* familial prostate cancer, *SPC* sporadic prostate cancer, *NOS* Newcastle-Ottawa Scale.

**Table 2:** The patients’ clinical data in all included articles.

First author of study and [Ref.]	Follow-up (month)	PCa Population	Primary treatment	Age (years)			PSA level (ng/ml)			Outcome
				Total	Yes FH	No FH	Total	Yes FH	No FH	
Hanus et al. [24]	NR	Localized PCa	EBRT	68	66	68	16.7	16.9	16.6	BCR
Ray et al. [25]	48	Localized PCa	EBRT ± NADT	NR	68	68.4	NR	8.1	7.7	BCR
Lee et al. [26]	91	Localized PCa	RP	NR	62	64	NR	7.2	7.8	BCR
Spangler et al. [27]	21	Localized PCa	RP	NR	NR	NR	NR	NR	NR	BCR
Roehl et al. [28]	47	Localized PCa	RP	NR	63 (Sibling pairs) 61 (Hereditary) 60 (High density family member)	61	NR	6.5 (Sibling pairs) 5.9 (Hereditary) 6.1 (High density family member)	5.9	BCR
Kupelian et al. [29]	65	Localized PCa	RP (*n* = 1971) RT (*n* = 2141)	≤65 (*n* = 2059) >65 (*n* = 2053)	≤65 (*n* = 358) >65 (*n* = 301)	≤65 (*n* = 1701) >65 (*n* = 1752)	≤4 (*n* = 399) >4 to ≤10 (*n* = 2427) >10 to ≤20 (*n* = 869) >20 (*n* = 417)	≤4 (*n* = 66) >4 to ≤10 (*n* = 406) >10 to ≤20 (*n* = 131) >20 (*n* = 56)	≤4 (*n* = 333) >4 to ≤10 (*n* = 2021) >10 to ≤20 (*n* = 738) >20 (*n* = 361)	BCR
Peters et al. [30]	60	Localized PCa	LDR ± ADT	NR	65	67	NR	<10 (*n* = 142) 10 to 20 (*n* = 37) >20 (*n* = 14)	<10 (*n* = 1132) 10 to 20 (*n* = 286) >20 (*n* = 132)	BCR
Bagshaw et al. [31]	71	Localized PCa	IMRT	68.2	67.4	68.5	6.3	6	6.4	BCR CSS OS
Westerman et al. [13]	119	Localized PCa	RP	63	62	64	6.1	6	6.2	BCR CSS OS
Thalgott et al. [32]	74.5	Localized PCa	RP	63.9	63.0 (FPC)	65.9 (SPC)	11.8	11.5 (FPC)	11.1 (SPC)	BCR CSS (FPC/SPC)
Lee et al. [6]	40.5	Localized PCa	RP	NR	62.7	65.6	7.4	7.75	7.38	BCR

*PCa* prostate cancer, *FH* family history, *EBRT* external beam radiotherapy, *ADT* androgen deprivation therapy, *NADT* neoadjuvant ADT, *RP* radical prostatectomy, *RT* radiation therapy, *LDR* low-dose radiotherapy, *FPC* familial prostate cancer, *SPC* sporadic prostate cancer, *BCR* biochemical recurrence, *CSS* cancer-specific survival, *OS* overall survival.

### Meta-analysis

#### Association between family history and biochemical recurrence-free survival

**Fig. 2:**
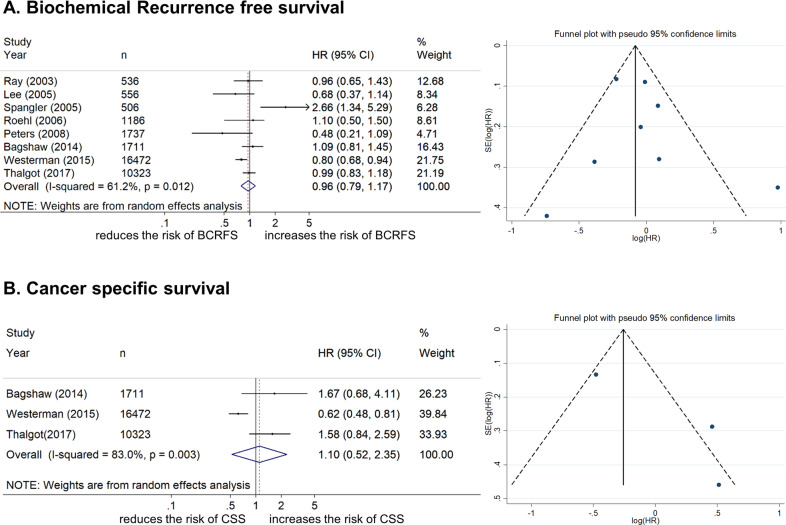
Forest and funnel plots showing the association of family history with oncological outcomes in localized prostate cancer patients. **A** Biochemical recurrence-free survival; **B** cancer-specific survival.

Eight studies including 33,027 patients provided data on the association of family history with biochemical recurrence (BCR). All patients had localized PCa and were treated with RP or RT. The forest plot (Fig. 2A) revealed that family history of PCa was not associated with BCR in localized PCa patients (pooled HR, 0.96; 95% CI, 0.79–1.17; *z* = 0.39). The Cochrane Q test (*c*^2^ = 18.02; *P* = 0.012) and I-square test (*I*^2^ = 61.2%) showed significant heterogeneity. The funnel identified one study over the pseudo 95% CI (Fig. 2A).

#### Association between family history and cancer-specific survival

Three studies including 28,506 patients provided data on the association of family history with cancer-specific mortality (CSM). The forest plot (Fig. 2B) showed that family history of PCa was not associated with CSS in localized PCa patients (pooled HR, 1.1; 95% CI, 0.52–2.35; *z* = 0.26). The Cochrane Q test (*c*^2^ = 11.74%; *P* = 0.003) and I-square test (*I*^2^ = 83.0%) showed significant heterogeneity. The funnel plot identified one study over the pseudo 95% CI (Fig. 2B).

### Subgroup analysis

#### Association between family history and BCRFS of prostatectomy

Four studies including 29,043 patients provided data on the association of family history with BCR of RP. All patients had localized PCa and were treated with RP. The forest plot (Fig. S1A) revealed that family history of PCa was not associated with BCRFS in localized PCa patients treated with RP (pooled HR, 0.99; 95% CI, 0.76–1.31; *z* = 0.04). The Cochrane Q test (*c*^2^ = 14.18; *P* = 0.007) and I-square test (*I*^2^ = 71.8%) showed significant heterogeneity. The funnel plot identified one study over the pseudo 95% CI (Fig. S1A).

#### Association between family history and BCRFS after radiation therapy

Three studies including 3984 patients provided data on the association of family history with BCR after RT. All patients had localized PCa and were treated with RT. The forest plot (Fig. S1B) revealed that family history of PCa was not associated with BCRFS in localized PCa patients treated with RT (pooled HR, 0.93; 95% CI, 0.67–1.30; *z* = 0.43). The Cochrane Q test (*c*^2^ = 3.46; *P* = 0.178) and I-square test (*I*^2^ = 42.1%) showed no significant heterogeneity. The funnel plot did not identify any publication bias (Fig. S1B).

## Discussion

To the best of our knowledge, our study is the first systematic review and meta-analysis investigating the impact of family history on oncological outcomes in localized PCa. In meta-analysis, we assessed data from eight published articles with a combined patient population of more than 33,000 patients. As only two studies evaluated the association of OS with family history, we excluded OS from the oncological outcomes in this meta-analysis. We found that family history of PCa did not increase the risk of BCR and CSM. In an additional sub-analysis, we evaluated the association of family history with BCR in patients treated by RP and RT, respectively, and found the same results as from the main analysis.

Although based on retrospective analyses with relatively small number of samples, some early reports suggested that patients with family history of PCa may experience more aggressive features of the disease [12, 33, 34]. However, our meta-analysis showed that oncological outcomes for patients with PCa did not differ substantially in the presence or absence of family history.

The clinical application of PSA screening is thought to be one of the main reasons for inconsistencies in these results. PSA screening was approved by the United States Food and Drug Administration in 1986 as a test to monitor patients with PCa, and in 1994 it was approved as a screening aid for diagnostic detection [35]. Some reports suggest that family history of PCa was an independent risk factor for aggressive oncological outcome in cohorts that did not receive PSA screening or that were studied before such screening became widely available, and that family history presented more favorable disease outcomes in PSA-screened cohorts [29, 36]. In addition, Lee et al. reported that, after the introduction of PSA testing, the risk of PCa diagnosis was significantly increased by a PCa diagnosis in a brother [37]. Because PCa develops relatively slowly, men with a positive family history of PCa are more likely to pay attention to their health and thus have a higher chance of detecting PCa in its early stages through PSA screening, which in turn produces a somewhat protective effect of family history. Indeed, family history is reported to be associated with a higher likelihood of PCa diagnosis at a younger age [2]; in our systematic review, the age of diagnosis was lower in PCa patients with a positive family history than in those without (Table 2).

On the other hand, Spangler et al. reported that family history of PCa was associated with higher tumor stage at diagnosis in men diagnosed before 60 years of age, and that men over 60 who had family history of PCa were significantly more likely to experience BCR [27]. Whether younger diagnostic age is related to earlier presentation for diagnosis because of increased familial awareness of the disease or whether the earlier age at diagnosis reflects a biological characteristic of familial PCa remains uncertain. However, with the availability of PSA screening, although the relative risk for PCa diagnosis doubled in first-degree male relatives of PCa patients [2], family history of PCa did not increase the risk of worse oncological outcome for PCa. These results indicate that PCa patients with a positive family history need not worry excessively about their oncological outcome if they receive regular PSA screening.

Although the present study represents the first systematic review and meta-analysis that evaluated the effects of family history of PCa on oncological outcomes, there are several limitations. In this study, articles published in other than English were excluded, increasing the risk of selection bias. Only non-randomized observational studies were included and all of them had a retrospective design. The studies differ in their methods for collecting family history data, with several studies relying on patient self-reporting and physician queries to determine the presence or absence of family history of PCa, increasing the risk of recall bias. In addition, although at the time of this writing the primary treatment options for localized PCa were RP, RT, androgen deprivation therapy, and active surveillance, only RP and RT were selected for our meta-analysis. The definition of family history is also a limitation. In most of the studies, the definition of family history was one or more first-degree relatives, but several recent reports discuss hereditary PCa, which suggests subgroups of familial PCa with a likelihood of genetic predisposition [3, 32, 38]. Few studies have assessed this particular patient group, and the evaluation of the risk of hereditary PCa may provide different results. The heterogeneity of germline genetic mutations in PCa was not also evaluated in this study. Further investigation will be required to elucidate the impact of these subtypes on oncological outcomes of PCa.

## Conclusion

This study is the first systematic review and meta-analysis investigating the impact of family history on oncological outcomes in localized PCa. Although several limitations were included, the results of this meta-analysis suggest that family history of PCa did not increase the risk of BCR or CSM in localized PCa patients. These results will be utilized for patient counseling.

## Supplementary information

Supplementary Figure 1

Supplementary Table 1

Supplementary Table 2
