# Supplementary figures and images for: Impact of family history on oncological outcomes in primary therapy for localized prostate cancer patients: a systematic review and meta-analysis
Source: Prostate Cancer Prostatic Dis. 2021 Feb 15;24(3):638–46. doi: 10.1038/s41391-021-00329-0 (PMC8384618; doi:10.1038/s41391-021-00329-0)

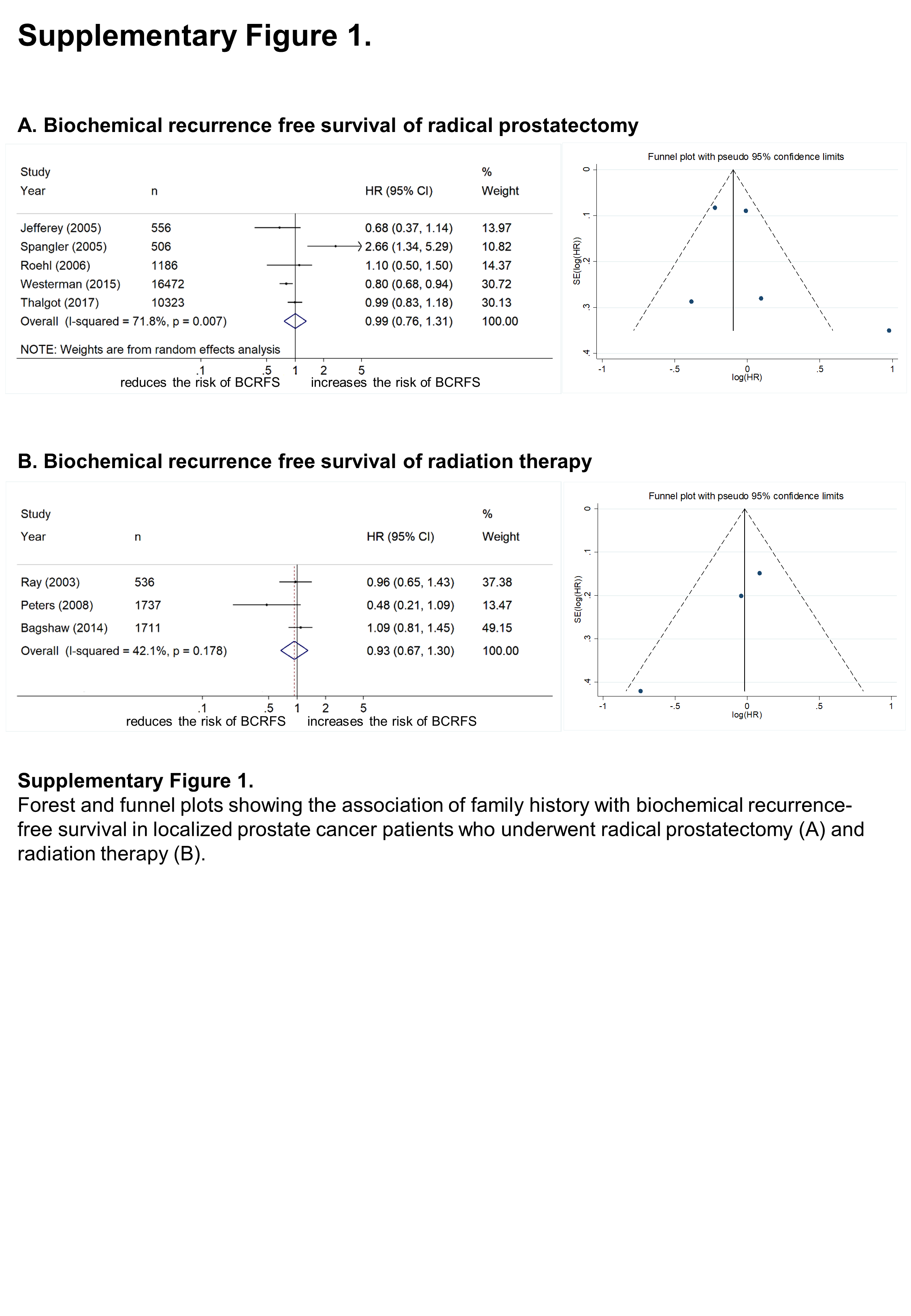

Supplement: Supplementary file 1 — Supplementary Figure 1 [file 41391_2021_329_MOESM1_ESM.tif]
