# Supplementary material for: Impact of family history on oncological outcomes in primary therapy for localized prostate cancer patients: a systematic review and meta-analysis
Source: Prostate Cancer Prostatic Dis. 2021 Feb 15;24(3):638–46. doi: 10.1038/s41391-021-00329-0 (PMC8384618; doi:10.1038/s41391-021-00329-0)
Supplement: Supplementary file 2 — Supplementary Table 1 [file 41391_2021_329_MOESM2_ESM.pdf]

Supplementary Table 1. Risk of bias assessment for individual studies using the Risk of Bias in Non-randomized Studies of Interventions tool (ROBINS-I).

Outcome: Biochemical recurrence	Risk of Bias							
Author	Confounding	Participant selection	Classification of interventions	Departures from intended interventions	Missing data	Measurement of outcomes	Selection of the reported results	Overall
Ray (2003)	moderate	moderate	moderate	Low	moderate	Low	Low	moderate
Lee (2005)	Low	moderate	serious	Low	Low	Low	moderate	serious
Spangler (2005)	moderate	Low	moderate	Low	moderate	Low	Low	moderate
Roel (2006)	serious	moderate	serious	Low	Low	moderate	moderate	serious
Peters (2008)	moderate	moderate	serious	moderate	moderate	Low	moderate	serious
Bagshaw (2014)	moderate	low	moderate	moderate	moderate	Low	Low	moderate
Westerman (2015)	Low	moderate	serious	Low	moderate	Low	moderate	serious
Thalgott (2017)	Low	Low	moderate	Low	moderate	Low	Low	moderate

Each domain was classiffied as low risk, moderate risk, serious risk, and critical risk or no information on which to base the judgement.

An overall bias assessment was performed usign the recommendated scale.
